# Occurrence of Gastrointestinal Parasites in Small Ruminants in the Central Part of Myanmar

**DOI:** 10.1155/2020/8826327

**Published:** 2020-11-25

**Authors:** Shwe Yee Win, Myintzu Win, Ei Phyu Thwin, Lat Lat Htun, Myint Myint Hmoon, Hla Myet Chel, Yu Nandi Thaw, Nyein Chan Soe, Thwe Thwe Phyo, Su Su Thein, Yadanar Khaing, Aye Aye Than, Saw Bawm

**Affiliations:** ^1^Department of Pharmacology and Parasitology, University of Veterinary Science, Yezin, Nay Pyi Taw, Myanmar; ^2^Department of Zoology, University of Magway, Myanmar; ^3^Department of International Relations and Information Technology, University of Veterinary Science, Yezin, Nay Pyi Taw, Myanmar; ^4^Department of Zoology, Kalay University, Myanmar

## Abstract

Gastrointestinal parasite infection in small ruminants remains one of the major economic losses caused by reduced productivity. A total of 380 faecal samples were taken from 280 sheeps in Magway and Pwintbyu Townships and 100 goats in Natmauk Township, Myanmar. Faecal flotation and sedimentation methods were carried out to detect the presence of parasitic infections. Faecal egg and oocyst counts were carried out using the McMaster technique. The overall occurrence of gastrointestinal parasites in small ruminants was 98.4% (374/380). The occurrence of gastrointestinal parasites in sheep (99.3%) was higher than that in goats (96%). The highest occurrence was found in *Eimeria* spp. (96%), followed by Trichostrongyle (77.1%), *Trichuris* spp. (35%), and *Moniezia expansa* (14%). The mixed infection rate was 84.8% (317/374), while a single infection was 15.2% (57/374). The mean eggs per gram (EPG) and oocysts per gram (OPG) of faeces were ranged from 50 to 600 and 50 to 29,800, respectively. Among the 4 nucleotide sequences isolated, one sequence was 94.10-94.47% similarity with *Trichostrongylus colubriformis*, reported from Laos, and three sequences showed 96.64-99.46% identity with *Haemonchus contortus* from Laos, China, India, and Mongolia. As gastrointestinal parasite infection in small ruminants was relatively high in the study area, the development of appropriate treatment and control measures should be provided to reduce production losses.

## 1. Introduction

Intestinal parasites have become more difficult to manage in small ruminants because of the parasite's increasing resistance to several anthelmintics [1]. Amongst livestock diseases, gastrointestinal (GI) parasite infection in ruminants results in adverse effects on feed intake, growth rate, carcass weight and composition, wool growth, fertility, and milk yield [2].

Another important parasitic infection in small ruminants is coccidiosis, which is caused by coccidian parasites of the genus *Eimeria*. It prevails prevalently in many parts of the world, either clinically or subclinically, and contributes to enteric disease, especially in young or stressed goats under poor farm management, leading to high mortality in goat kids [3]. Moreover, coinfection with other Trichostrongyle nematodes makes for diagnoses clinically coccidiosis difficult [4]. Parasitic diseases negatively impact not only direct losses related to acute illness and death and damage, condemnation of organs, and cost of veterinary service but also indirect losses, including decreases in productive potentials, such as decreased growth rate, weight loss in young growing animals, and late maturity of slaughter stock [5].

Small ruminant production holds an important sector for the development of socioeconomic status in developing countries and supports a variety of socioeconomic functions throughout the world. In Myanmar, the small ruminant population was about 2.1 million in 2018 (2018 census of Livestock Breeding and Veterinary Department, Myanmar). Small ruminants are raised with systems of semi-intensive and free-ranging in grazing land in the central part of Myanmar because of the relatively low rainfall in the area. Currently, farmers are facing a lot of problems in livestock production due to climate changes, nutritional insufficiency, and infectious pathogens, including bacteria, viruses, and parasites. The great economic losses due to GI nematode infection, especially the Trichostrongyle nematodes, have more impact on sheep production than goat production.

Keeping in view the importance of gastrointestinal parasites in small ruminants, this study was conducted to investigate the occurrence of gastrointestinal parasites and determine the species of parasites in the Magway, Pwintbyu, and Natmauk Townships, situated in the central dry zone (CDZ) of Myanmar. Moreover, this study is aimed at genetically characterizing the Trichostrongyle nematodes causing economic losses in sheep production in Myanmar.

## 2. Materials and Methods

### 2.1. Study Area and Study Design

**Figure 1:**
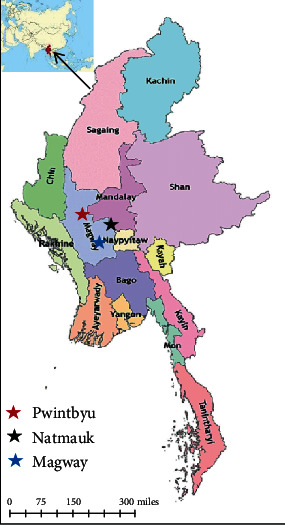
Location map of the study area.

In this study, samples were collected from Natmauk, Magway, and Pwintbyu Townships (Figure 1). Natmauk Township is situated at 19°13′0^″^N latitude and 95°36′0^″^E longitude and 171.92 m elevation above sea level. Magway Township is situated at 20°9′15.95^″^N latitude and 94°56′43.73^″^E longitude and elevated at 83.38 m above sea level. Pwintbyu Township is situated at 20°22′22.07^″^N latitude and 94°40′24.44^″^E longitude and 59.2 m elevation above sea level. The study areas are located in the central part of Myanmar and are included in the central dry zone (CDZ). Sheep and goat farming is popular in the central part of the country because this area has lower rainfall when compared to the other parts of the country. Moreover, government and nongovernment organizations focused on livestock farming, especially on small ruminants for socioeconomic development in CDZ areas.

### 2.2. Farm Management Practices

The animals with a flock size ranging from 10 to 30 were owned by small-scale holder farmers. Animals were kept under extensive management systems, where they were allowed free ranging on farmland during the day with minimum supplementation of concentrates from groundnut and sesame cake. Veterinary care is little or not provided in these areas. The animals were farmed on common grazing areas that had indiscriminately contacted other animal species.

### 2.3. Sample Collection

A total of 380 faecal samples, 280 sheep samples from Magway and Pwintbyu Townships and 100 goat samples from Natmauk Township, were randomly collected (Table 1). Faecal samples were directly collected from the rectum of sheep and goats using latex gloves per animal to avoid contamination. The collected faecal sample was put in a labelled zip-lock bag, kept in an icebox, and carried to the Laboratory of Department of Pharmacology and Parasitology to examine the helminths egg and protozoa oocyst. The samples were kept in a refrigerator at 4°C before the faecal examination was performed.

### 2.4. Questionnaire Survey

During sample collection, the facts concerning with the animal husbandry practices such as feeding practice, farm management, veterinary care, and anthelmintic treatment were asked to the farm owners. Visual observation was also carried out to know the presence or absence of an abnormal clinical appearance of the sampled animal. Moreover, breed, age, and sex were recorded. All the collected goats were female.

### 2.5. Faecal Examination

Faecal flotation and sedimentation were carried out according to Foreyt [6]. For faecal flotation, 5 g of the faecal sample was mixed with 45 ml of water in a beaker and filtered through a sieve into a second beaker. The solute in the second beaker was put in a 15 ml centrifuge tube and centrifuged at 1500 rpm for 10 mins. Then, the supernatant fluid was discarded. Next, the saturated sugar solution was added into the tube and centrifuged again at 1500 rpm for 10 mins. After that, the tube was filled with a sugar solution; a coverslip was placed on the tube and placed on a glass slide for microscopic examination after 30 mins of waiting. For the sedimentation method, 5 g of faeces was mixed with 200 ml of water in a beaker and poured the mixture into a new beaker through a sieve. After 10 mins, approximately 70% of the supernatant fluid in the beaker was discarded and refilled the beaker with water. This step was repeated 3-5 times until the supernatant fluid was clear. Approximately, 90% of the supernatant fluid was discarded. Finally, one drop of the sediment was placed on the glass slide, and a coverslip was placed on the glass slide and examined under a microscope.

For the McMaster method, 3 g of faeces was mixed in 15 ml of water and filtered through a sieve. The solute was poured into a 15 ml tube and centrifuged at 1500 rpm for 10 mins. The supernatant fluid in the tube was discarded and filled with a saturated sugar solution. The tube was centrifuged at 1500 rpm for 10 mins. Then, the tube was stood with a rank; the sugar solution was filled with a pipette and waited for 30 mins to float the parasite eggs and oocysts. The supernatant fluid was transferred with a pipette into both sides of a McMaster counting chamber. The number of eggs or oocysts in both chambers was counted and multiplied by 50 for the total number of either eggs or oocysts per gram of faeces. According to Soulsby [7], faecal egg count (FEC) values were classified as free, low (<500 EPG), medium (500–1000 EPG), and high (>1000 EPG). Similarly, faecal oocyst count (FOC) values were classified into *Eimeria*-free, low (<1800 OPG), medium (1800–6000 OPG), and high (> 6000 OPG) according to Idris et al. [8]. In this study, the McMaster counting method was performed individually in all the goat faecal samples, whereas in sheep, only one out of ten positive samples was done for egg and oocyst counting. To identify the size of the parasite eggs, measurements were performed with an ocular micrometre. The typical sizes of Trichostrongyle, *Trichuris* spp., *Moniezia expansa*, and *Eimeria* spp. were 85 × 40 *μ*m, 75 × 35 *μ*m, 50 × 60 *μ*m, and 16 − 47 × 14 − 32 *μ*m, respectively, [6].

### 2.6. Molecular Characterization of Trichostrongyle Nematodes

DNA extraction and PCR conduction were carried out at the Laboratory of Department of Pharmacology and Parasitology, University of Veterinary Science, Yezin, Nay Pyi Taw, Myanmar. To know the genetic information of Trichostrongyle nematode, which has a greatly impact on the sheep production, five representative microscopically positive samples were used for DNA extraction with a DNA extraction kit (QIAamp Fast DNA Stool Mini Kit, Qiagen, Germany) following the manufacturer's instructions. The ITS region of the nematode was amplified using universal nematode primers, NC2 (5′-TTAGTTTCTTTTCCTCCGCT-3′) and NC5 (5′-GTAGGTGAACCTGCGGAAGGATCAT T-3′) described by Newton et al. [9]. PCR was carried out with the total reaction volume containing 12.5 *μ*l of 2× Gflet buffer, 0.5 *μ*l of each of 10 *μ*M forward and reverse primers, 0.5 *μ*l of DNA polymerase, 10 *μ*l of deionized sterile water, and 1 *μ*l of sample DNA using a Thermal cycler (PCR Max, Thermo Fisher Scientific) with the following conditions: initialization at 94°C for 5mins, followed by denaturation at 98°C for 10 sec, annealing at 55°C for 15 sec, extension at 68°C for 1 min, and then final extension at 68°C for 5mins for 40 cycles. PCR products were conducted to 1% agarose gel electrophoresis for 30 mins at 100 V, and the positive bands were visualized at ~1000 bp. PCR product purification and sequencing were carried out at Hokkaido University, Japan. The obtained nucleotide sequences were analyzed using the NCBI BLAST program (https://blast.ncbi.nlm.nih.gov/Blast.cgi) to match the highly similar sequences and conducted for phylogenetic analysis. Sequence data from this study were deposited in GenBank (MT568603-MT568606).

### 2.7. Statistical Analysis

The animal was considered as a positive when one or more parasite eggs or oocysts were found in the faecal examination. A chi-square test was used to assess the association between the occurrence of gastrointestinal parasites and age, sex, and species of animals using SPSS software version 20.0. The level of significance was considered at *P* < 0.05.

## 3. Results

### 3.1. Microscopic Detection of GI Parasites in Sheep and Goats

**Figure 2:**
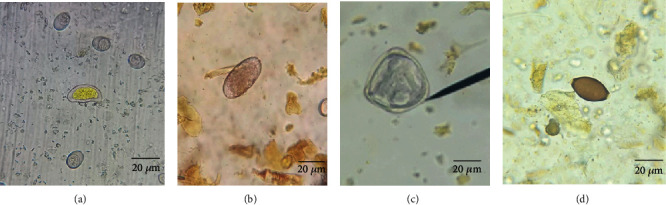
Parasite oocyst/eggs of sheep: (a) *Eimeria* spp. (×400), (b) Trichostrongyle (×100), (c) *Moniezia expansa* (×400), and (d) *Trichuris* spp. (×400).

**Figure 3:**
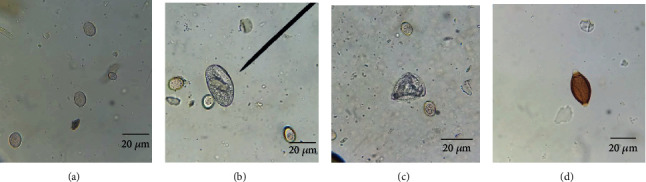
Parasite oocyst/eggs of goat: (a) *Eimeria* spp. (×400), (b) Trichostrongyle spp. (×400), (c) *Moniezia expa*nsa (×100), and (d) Trichuris spp. (×400).

Out of 380 faecal samples examined, 374 samples were positive for gastrointestinal parasites, with an overall occurrence of 98.4%. The occurrences of gastrointestinal parasites in sheep and goats are shown in Table 2. There was a significant difference in the occurrence of GI parasites between sheep and goats. Four gastrointestinal parasite species (*Eimeria* spp., Trichostrongyle, *Trichuris* spp., and *Moniezia expansa*) were observed (Figures 2 and 3). Mixed infestation (84.8%, 317/374) was more dominant than single infestation (15.2%, 57/374). However, any clinical signs were not found in all of the sampled animals during sampling.

The FEC/FOC ranges of sheep and goats are shown in Table 3. Although all sampled sheep and goats showed a low infection rate with nematode and cestode, the high infection rate was recorded in *Eimeria* spp. The measurements of the eggs/oocysts of GI parasites in sheep and goat were consistent with the standard measure and are shown in Table 4.

The occurrence of gastrointestinal parasites according to the age and sex of sheep and goats is shown in Table 5. There was an association between the occurrence of Trichostrongyle spp. and *Moniezia expansa* and the age of sheep. However, no association was observed between the occurrence of parasites and the age group of goats. According to the sex group of sheep, there was an association with the occurrence of Trichostrongyle nematodes.

### 3.2. Molecular Characterization of Trichostrongyle Nematodes in Sheep

**Figure 4:**
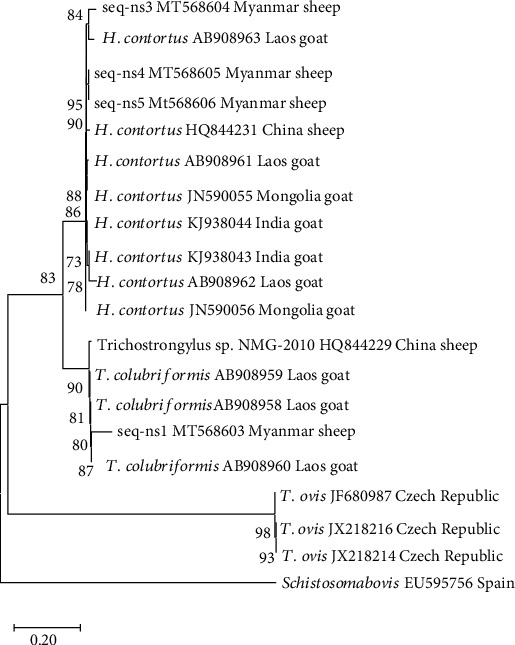
Phylogenetic analysis of ITS sequences of Trichostrongyle nematode by the Neighbor-Joining method using MEGAX. Numbers indicate bootstrap percentages (1000 replicates). The scale indicates the divergence time.

For molecular characterization of the Trichostrongyle nematode of sheep, the representative five samples were conducted to conventional PCR, and the positive bands were visualized at ~1000 bp. The sharp bands were cut off and carried out to purification and sequencing analysis. The total of 4 out of the 5 obtained homologues showed good signals and were used for phylogenetic analysis. After matching with the resultant sequences and BLAST sequences, the sequence seq-ns1 showed 94.10-94.47% similarity with *T. colubriformis*, reported from Laos (AB908959, AB908958, and AB908960) and China (HQ8442291) and clustered in the same clade. The sequence seq-ns3, seq-ns4, and seq-ns5 showed 94.10-94.47% identity with *H. contortus* (AB908961, AB908962, AB908963, HQ844231, KJ938043, KJ938044, JN590055, and JN590056), reported from Laos, China, India, and Mongolia. The seq-ns4 clustered together with seq-ns5 in the same clade. The NJ tree showed that the 4 nucleotide sequences belonged to Trichostrongylus spp. and Haemonchus spp. of the family Trichostrongylidae from neighboring countries (Figure 4).

## 4. Discussion

Gastrointestinal parasite infection can cause a major problem in small ruminant production, especially in tropics and subtropics. Moreover, GI parasite infection can cause severe economic losses through a reduction in food intake, weight gain, fertility rate, increased treatment costs, and mortality in heavily parasitized animals [10].

In this study, a high infection rate of GI parasites was exhibited in small ruminants, with an overall occurrence of 98.4%. This finding is consistent with the occurrence of GI parasites in small ruminants in India (91-95%) [11, 12] and cattle and small ruminants in Ghana (90.8%) [13]. However, a lower occurrence was observed in Nigeria (69.64%) [1]. The high occurrence of GI parasites in sheep and goats might be related to hygienic practices on farms and management of grazing land. The possible way of infection might be due to contamination of the parasite in the pasture since the parasite species found in this study were infected via by ingestion of contaminated food. Moreover, there was no association (*P* > 0.05) between the occurrence of GI parasites and deworming practices on farms in this study. Therefore, it could be assumed that GI parasites have become resistant to anthelmintics being used. The prevailing agroclimatic conditions like overstocking and grazing of young and adult animals together supply an ideal condition for the transmission of GI parasites [14].

The results of the species-wise prevalence revealed that the sheep were more susceptible to helminths infection (99.3%) than goats (96%). Similar findings were reported in the western zone of India with the occurrence of 85.16% and 79.24% in sheep and goats, respectively, [15]. In contrast, the occurrence of GI parasite prevalence was higher in goats than that in sheep in West India (98% and 88%) [11], Pakistan (78.2% and 78%) [16], and Bangladesh (77% and 65.9%) [17]. The higher prevalence of GI parasitic infections in sheep as compared to goats was probably due to their grazing behavior. Sheep grazes close to the ground, so the risk of ingestion of parasitic ova is comparatively higher than that of goats, as they are browsers [18]. In the present study, this variation might be due to differences in geographical conditions of the study area and rearing and management practices related to the nutritional status of animals.

A high rate (84.8%) of mixed GI parasite infection was observed, while a single infection was 15.2%. A similar finding was discovered in India with the occurrence of 92% in mixed infestation and 8% in single infestations [11]. This might be due to the fact that having of anyone parasite species infestation would lead to a greater chance for more infections.

High infection of *Eimeria* spp. (95.8%) was observed in this study, followed by Trichostrongyle, *Trichuris* spp., and *Moniezia expansa* with the occurrence of 77.1%, 35%, and 13.9%, respectively. In India, *Haemonchus contortus* showed the highest infection rate (43.7%), followed by *Eimeria* spp. (15.40%), *Paramphistomum* spp. (8.91%), *Trichuris* spp. (8.72%), *Strongyloides* spp. (4.16%), *Fasciola gigantica* (4.16%), and *Moniezia expansa* (3.39%) [12]. The factors responsible for variations in the prevalence of different parasitic diseases might be the different climate and immune status of the individual animal.

Although a low FEC and a high FOC range were observed, any apparent clinical signs were not found in sampled local breed sheep and goats. This could be explained that local breeds have acquired strong immunity to infection of GI parasites due to recurrent infections. Moreover, FEC could be affected by factors such as study area, season, and age, as shown by Zvinorova et al. [19].

The occurrence of *Eimeria* spp. (*P* = 0.016), Trichostrongyle (*P* ≤ 0.001), *Trichuris* spp. (*P* = 0.007), and *Moniezia expansa* (*P* = 0.004) were found significantly related to host species. This relationship might be due to differences in feeding behavior of host species and immune status of individual animals.

Although the overall occurrence of GI parasites in sheep and goat was numerically higher at <6 months of age than at <6 months of age, there was no significant association between the occurrence and age factors. However, there was an association between the occurrence of Trichostrongyle and *Moniezia expansa* and the age of sheep. In goats, a significant association was observed between the occurrence of Trichostrongyle and age groups. This might be due to less development of the immune system in young sheep, which could lead to susceptibility to infection. Likewise, lower occurrence in older sheep might be attributed to body resistance as they might have developed immunity due to repeated natural infections [20].

This study revealed that the occurrence was numerically higher in females (99.6%) than that in males (98.2%). Although there was no association between the occurrence of *Eimeria* spp., *Trichuris* spp., and *Moniezia expansa*, and sex, a significant association was observed between the occurrence of Trichostrongyle and sex of animals. However, Adua and Hassan [21] reported that gender showed no direct influence on the epidemiology and distribution of GI parasites among sheep and goats.

The molecular characterization of GI parasites in small ruminants is very limited information in Myanmar. Recently, Bawm et al. [22] reported the first detection of the three species of *Eimeria* (*E. christenseni*, *E. hirci*, and *E. arloingi*) using both microscopic and molecular methods in goats in Myanmar. In this study, the most common nematode, Trichostrongyle in sheep, was carried out for molecular characterization based on the amplification of the ITS region of DNA because we could not be sure of particular species of Trichostrongyle by microscopic examination, and their impact are affected on the great economic losses in sheep production. The ITS region contains reliable genetic markers to distinguish closely related species and is also used for the identification of strongylid nematodes to species [9]. The phylogenetic tree suggests that a shared evolutionary history has been mentioned between sheep and goats and therefore, they may have evolved from one common ancestor. Moreover, the nucleotide of these species is likely to be shared with species from neighboring countries because of the small ruminant border trade between Myanmar and neighboring countries.

## 5. Conclusion

Small ruminants in Myanmar were infected by a variety of GI parasites, the majority of which were coinfections. Although the occurrence of GI parasites was relatively high, any clinical signs were not found in the sampled animals. *Eimeria* spp. showed the highest infection rate, followed by Trichostrongyle, *Trichuris* spp., and *Moniezia expansa*. As the parasite intensity was relatively high in the study area, appropriate treatment and control measures should be provided. Further, molecular studies should be performed to know the genetic differences in GI parasites between Myanmar and other countries.

## Figures and Tables

**Table 1:** Total number of samples collected from three townships.

Species	Location	No. of collected samples
Sheep	Magway	140
Sheep	Pwintbyu	140
Goat	Natmauk	100
Total		380

**Table 2:** Occurrence of gastrointestinal parasites in sheep and goats.

Animal species	No. of examined	Positive no. (%)	No. (%) of parasite spp. observed			
			*Eimeria*	Trichostrongyle	*Trichuris*	*Moniezia expansa*
Sheep	280	278 (99.3)	273 (97.5)	244 (87.1)	109 (38.9)	30 (10.7)
Goat	100	96 (96)	91 (91)	49 (49)	24 (24)	23 (23)
Total	380	374 (98.4)	364 (95.8)	293 (77.1)	133 (35)	53 (13.9)
*p* value		0.044^∗^	0.016^∗^	0.000^∗∗^	0.007^∗∗^	0.004^∗∗^

^*^Level of significance (*P* < 0.05); ^**^level of significance (*P* < 0.01).

**Table 3:** Faecal eggs and oocyst counts (FEC/FOC) from sheep and goats.

Animal species	Range of FEC/FOC			
	*Eimeria*	Trichostrongyle	*Trichuris*	*Moniezia expansa*
Sheep	7350-29800	50-500	50-600	50-500
Goat	50-12250	50-400	50-100	50-100

**Table 4:** Measurement of GIT parasite eggs and oocyst of sheep and goats.

Parasite species	Measurement of length and width of parasite egg and oocyst of sheep and goat			
	Sheep		Goat	
	Length	Width	Length	Width
Trichostrongyle	70-80 *μ*m	30-35 *μ*m	80-85 *μ*m	35-40 *μ*m
*Trichuris* spp.	65-70 *μ*m	25-30 *μ*m	60-70 *μ*m	25-30 *μ*m
*Moniezia expansa*	60 *μ*m	50 *μ*m	55-60 *μ*m	50 *μ*m
*Eimeria* spp.	15-35 *μ*m	10-25 *μ*m	30 *μ*m	10-15 *μ*m

**Table 5:** Description of gastrointestinal parasites according to age and sex of sheep and goats.

Category	No. of examined	Total positive no. (%)	Positive no. (%)			
			*Eimeria*	Trichostrongyle	*Trichuris*	*Moniezia expansa*
Sheep						
Age						
>6months	195	193 (99)	191 (97.9)	180 (92.3)	78 (40.0)	14 (7.2)
<6months	85	85 (100)	82 (96.4)	64 (75.2)	31 (36.4)	16 (18.8)
*P* value		1.000	0.437	0.000^∗∗^	0.597	0.006^∗∗^
Sex						
Male	57	57 (100)	56 (98.2)	45 (78.9)	28 (49.1)	6 (10.5)
Female	223	221 (99.1)	217 (97.3)	199 (89.2)	81 (36.3)	24 (10.7)
		1.000	1.000	0.047^∗^	0.094	1.000
Goat						
Age						
>6months	37	34 (91.8)	32 (86.5)	22 (59.4)	10 ((27)	6 (16.2)
<6months	63	62 (98.4)	59 (93.7)	27 (42.8)	14 (22.2)	17 (27)
*P* value		0.142	0.073	0.147	0.632	0.623

^*^level of significance (*P* < 0.05). ^**^level of significance (*P* < 0.01).

## Data Availability

Sequence data from this study were deposited in GenBank (MT568603-MT568606).
